# Fuzzy Optimized Attention Network with Multi-Instance Deep Learning (FOAN-MIDL) for Alzheimer’s Disease Diagnosis with Structural Magnetic Resonance Imaging (sMRI)

**DOI:** 10.3390/diagnostics15121516

**Published:** 2025-06-14

**Authors:** Afnan M. Alhassan, Nouf I. Altmami

**Affiliations:** Department of Computer Science, College of Computing and Information Technology, Shaqra University, Shaqra 11961, Saudi Arabia

**Keywords:** Alzheimer’s disease (AD), attention mechanism, fuzzy salp swarm algorithm (FSSA), multi-instance deep learning (MIDL), convolutional neural network (CNN), structural magnetic resonance imaging (sMRI)

## Abstract

**Background/Objectives:** Alzheimer’s disease (AD) is the leading cause of dementia and is characterized by progressive neurodegeneration, resulting in cognitive impairment and structural brain changes. Although no curative treatment exists, pharmacological therapies like cholinesterase inhibitors and NMDA receptor antagonists may deliver symptomatic relief and modestly delay disease progression. Structural magnetic resonance imaging (sMRI) is a commonly utilized modality for the diagnosis of brain neurological diseases and may indicate abnormalities. However, improving the recognition of discriminative characteristics is the primary difficulty in diagnosis utilizing sMRI. **Methods**: To tackle this problem, the Fuzzy Optimized Attention Network with Multi-Instance Deep Learning (FOA-MIDL) system is presented for the prodromal phase of mild cognitive impairment (MCI) and the initial detection of AD. **Results:** An attention technique to estimate the weight of every case is presented: the fuzzy salp swarm algorithm (FSSA). The swarming actions of salps in oceans serve as the inspiration for the FSSA. When moving, the nutrient gradients influence the movement of leading salps during global search exploration, while the followers fully explore their local environment to adjust the classifiers’ parameters. To balance the relative contributions of every patch and produce a global distinct weighted image for the entire brain framework, the attention multi-instance learning (MIL) pooling procedure is developed. Attention-aware global classifiers are presented to improve the understanding of the integral characteristics and form judgments for AD-related categorization. The Alzheimer’s Disease Neuroimaging Initiative (ADNI) and the Australian Imaging, Biomarker, and Lifestyle Flagship Study on Ageing (AIBL) provided the two datasets (ADNI and AIBL) utilized in this work. **Conclusions:** Compared to many cutting-edge techniques, the findings demonstrate that the FOA-MIDL system may determine discriminative pathological areas and offer improved classification efficacy in terms of sensitivity (SEN), specificity (SPE), and accuracy.

## 1. Introduction

As the most prevalent form of dementia, AD results in persistent brain damage as well as gradual progressive and irreversible cognitive decline. To stop the progression of AD in the early period, medication must be started immediately. The primary basis for diagnosing AD at multiple stages is clinical. The severity of cognitive impairment determines its classification into three stages: preclinical, mild, and dementia. Periodic short-term progressive and irreversible cognitive decline with comparatively spared long-term memory is the first sign. sMRI is recognized as a common imaging tool in determining the phase of neurodegeneration because it is a non-contact diagnostic technique [1,2]. Numerous studies have demonstrated that sMRI-driven volume measurements, namely in the medial temporal lobe and hippocampal regions, can be utilized to predict AD development.

Although MRI is inexpensive and widely available, earlier attempts to distinguish between healthy aging and AD using the traditional sMRI-based AD diagnosis techniques typically divided the whole MR image into numerous areas with varying scales to improve the feature extraction of local irregular changes in brain structure [3]. It is separated into three levels: (1) the patch level, (2) the region level, and (3) the voxel level. The tissue characteristics taken from sMRI scans lead to the curse of dimensionality in voxel-level approaches [4]. Segmented regions of interest (ROI) are utilized in region-level approaches [5] to distinguish AD patients from regular controls to mitigate this issue. Nevertheless, these techniques for the segmentation of ROIs are resource-intensive. Accordingly, patch-level feature representations [6] are suggested as a more efficient way to describe the local structural alterations in sMRI images. Patch-level approaches still face difficulties in integrating the local patches into a global depiction of features for the entire brain system.

Patch-level techniques rely on semi-automated segmentation techniques with important drawbacks, particularly small sample sizes. This has spurred the development of increasingly complex machine learning (ML)-based techniques for the analysis of MRI data. Many artificial intelligence (AI) methods, such as traditional voxel-based ML and DL-based techniques, have been implemented to support cognitive diagnosis when analyzing brain sMRI images [7,8,9]. An increasing body of research utilizes DL to evaluate sMRI images by training a complete algorithm, without the need for custom features, based on advancements in DL—particularly the effective employment of CNNs in the past decade [10,11,12].

The present CNN classifiers might not incorporate all disease-related shrinkage patterns dispersed throughout the brain and fail to adequately compensate for individual variances within the same template space. When classifying images in CNNs, it is usually assumed that each image has a different label. A single class cannot be used for an entire human disease image since the image may display several illness features in real medical operations. MIL is the term utilized to describe this common problem. The MIL approach to medical image analysis is employed since, typically, medical image datasets are small, with weak labeling but a reasonably high resolution [13]. The merging of machine learning and models of neurons has grown popular due to the rapid advancements in DL [14].

The existing studies do not completely describe the underlying rationale; instead, they give an overview of efforts to merge CNNs and MIL. An attention-based approach that increases MIL’s flexibility and interpretability was suggested by Ilse et al. [15]. Since then, there has been much interest in research on attention-based MIL. Attention-driven deep MIL for whole-slide image classification was suggested by Yao et al. [16]. An attention-based time-incremental CNN for multi-class identification was presented in [17] to achieve temporal and spatial data fusion from electrocardiograms.

This paper introduces the FOA-MIDL network for the early detection and prodromal stage of MCI. The FSSA is presented, with attention utilized to calculate the relative importance of every occurrence. The swarming habits of salps in oceans serve as the inspiration for the FSSA. As they move, nutrient gradients influence the movement of the leading salps during global search exploration, while others perform thorough local exploration to fine-tune the classifiers’ parameters. To balance the relative contributions of every patch and produce a globally distinct weighted image for the entire brain framework, the attention MIL pooling (A-MIDL) procedure is suggested. According to our research findings, the suggested approach outperforms numerous modern techniques regarding its detection effectiveness and can detect discriminative problematic areas in sMRI images.

## 2. Literature Review

Lama et al. [18] proposed an AD detection method that employs SVM, IVM, and RELM to distinguish between AD, MCI, and HC individuals. Important feature vectors are chosen utilizing the greedy score-based feature selection method. Furthermore, a discriminative technique centered on kernels handles intricate data distributions. They examined these classifiers’ results utilizing volumetric sMR image data from ADNI datasets. This research utilizing ADNI data demonstrates that feature selection in conjunction with RELM can greatly increase the classification precision for AD in patients with MCI and HC.

Li et al. [19] proposed that DenseNets can become familiar with the different local characteristics of MR brain images, which can be merged to classify AD. Initially, the entire brain image is divided into distinct local regions; then, many 3D patches are extracted from every area. Secondly, the K-Means algorithm is employed to cluster the patches from each region into distinct groups. Thirdly, the patch properties for each cluster are learned by building a DenseNet, and the attributes from each region’s discriminative clusters are combined for classification. Ultimately, the final image categorization is improved by combining the classification outcomes from many local locations. To address classification disputes, the suggested technique may progressively learn MRI characteristics from the local patch level to the global image level. 

Cui and Liu [20] suggested an AI method for the analysis of the hippocampal area that combines shape analysis and a 3D highly interconnected CN to associate the hippocampus’s local and global data in identifying AD. The suggested strategy can utilize global and local visual features to improve the categorization. It does not require tissue segmentation. A comparison of the outcomes shows that the suggested approach outperforms others. An automated ML technique for the identification of people with early and late MCI, AD, and cognitively normal aging was created by Rallabandi et al. [21]. The SVM-RBF demonstrated good classification accuracy across dementia stages. ML techniques are advantageous for radiological imaging activities like detection and risk evaluation.

Wang et al. [22] suggested a collection of 3D highly interconnected convolutional networks for 3D MRI-based AD and MCI detection. Dense connections were implemented to optimize the data flow, where every layer had a direct link with every other layer. Bottleneck and transition layers were utilized to lower the parameters and create more compact simulations. Next, 3D-DenseNets with various designs were combined utilizing the probability-based fusion technique. In-depth tests examined the 3D-DenseNet’s efficiency under various hyperparameters and structure configurations. The ADNI dataset proved the suggested algorithm’s higher accuracy.

Zhao et al. [23] proposed the application of sMRI because of its low cost. They studied key components of several AI methods related to AD, compiled findings from various research teams, analyzed the difficulties at hand, and suggested potential areas of study. Overall, DL methods outperform typical ML methods in discovering AD patterns because they aim to uncover hidden representations and connections across diverse parts of the scanned images. Eventually, they could be used to create a diagnostic system utilized for various forms of dementia in the future. AbdulAzeem et al. [11] proposed a methodology for AD categorization utilizing a CNN. During the digital image processing phase, adaptive thresholding is employed. The innovative method utilizes the outdated thresholding operator, which sets a global threshold over all pixels. Adaptive thresholding modifies the threshold constantly in response to variations in the image. This can adapt to the image’s changing lighting conditions. The optimization procedure takes advantage of the Adam optimizer. Applying the Adam optimizer to AD images results in faster convergence.

Chen and Xia [24] proposed an ISDL framework for crucial brain area detection and combined deep feature extraction for AD and MCI diagnosis. A DFE is first established to collect the local-to-global structural data produced from 62 cortical areas. To eliminate unnecessary cortical regions from the method of diagnosis, a sparse regression is then created to determine the key cortical regions and integrated into the DFE component. The two modules’ variables are updated alternately. According to the authors’ findings, the ISDL system offers an advanced approach to AD-CN categorization and MCI-to-AD diagnosis.

Feng et al. [25] proposed a technique to develop an NCSIN in the frequency domain to detect correlations among the anomalous energy distribution patterns associated with AD. In particular, downsampling and reconstruction processes modify a 2D image of the preprocessed sMRI images. Next, the 2D image is subjected to nonsubsampled contourlet transforms to produce directional subbands. On a single scale, each directional subband is represented by a CV, which is believed to be an NCSIN node. The edge that connects any two nodes is then weighted with CS. A network feature of the sMRI image is utilized for AD categorization by concatenating the edge and node characteristics.

Zhu et al. [26] proposed the DA-MIDL framework to identify MCI and AD. There are three main parts in DA-MIDL, and the objective is to examine the characteristics of unusually altered microstructures by extracting distinctive traits from every sMRI patch. The steps are as follows: (1) employ PatchNets with spatial attention blocks; (2) employ an A-MIDL function to balance the relative contributions and produce a global distinct weighted image for the brain form; and (3) employ attention-aware global classifiers to acquire more integral structures and formulate decisions associated with AD categorization. DA-MIDL was assessed based on 1689 participants’ baseline sMRI scans from two separate datasets.

Yuto Uchida et al. [27] discussed the microstructural neurodegeneration of the entorhinal–hippocampus pathway along the AD continuum. An 11.7T diffusion MRI instrument was used to scan postmortem brain specimens from non-AD, preclinical AD, and AD dementia patients. Serial histological tests confirmed the myelinated fiber and neuronal cell statuses after imaging. The entorhinal layer II islands and perforant route fibers could be recognized at a 250 m (zipped to 125 m) isotropic resolution in non-AD and preclinical AD patients but not in AD dementia patients after histological confirmation. Entorhinal layer II had the greatest FA value in non-AD and preclinical AD cases, whereas AD dementia patients had homogeneously low FA values. The FA values and perforant route fibers declined with AD (non-AD > preclinical AD > AD dementia).

Yuto Uchida et al. [28] discussed the acceleration of brain atrophy and progression from normal cognition to MCI. The BIOCARD study, which began at the NIH on 1 January 1995 and ended on 31 December 2005, and then continued at Johns Hopkins University on 1 January 2015 and ended on 31 October 2023, provided the data used in this cohort analysis. Cognitive testing revealed no abnormalities in any of the subjects. Included in the analysis were individuals for whom structural MRI of the brain and cerebrospinal fluid (CSF) measurements were available for more than a decade.

Diagnostic decision systems and medical image analysis have benefited greatly from swarm intelligence and bio-inspired optimization algorithms’ capacity for global search and adaptation. Feature selection, hyperparameter tweaking, and classification are some of the tasks in which notable algorithms like ant colony optimization (ACO), particle swarm optimization (PSO), and the grey wolf optimizer (GWO) have been implemented in neuroscience research. The original salp swarm algorithm (SSA) showed potential in optimizing the balance between exploration and exploitation; it was inspired by the swarming behavior of salps in marine chains. On the other hand, methods to deal with uncertainty and dynamic adaptability are absent from the conventional SSA. Some expansions that aim to overcome these restrictions include the adaptive SSA, Lévy flight SSA, and chaotic SSA. Extending this idea, the fuzzy salp swarm algorithm (FSSA) incorporates fuzzy logic to simulate uncertain environments and adjust salp motion on the fly. This enables finer-grained parameter tweaking and local searches in high-dimensional areas. Because of this, the FSSA is well suited to improving DL-based classifiers in structural MRI data to diagnose Alzheimer’s disease.

## 3. Proposed Methodology

**Figure 1:**
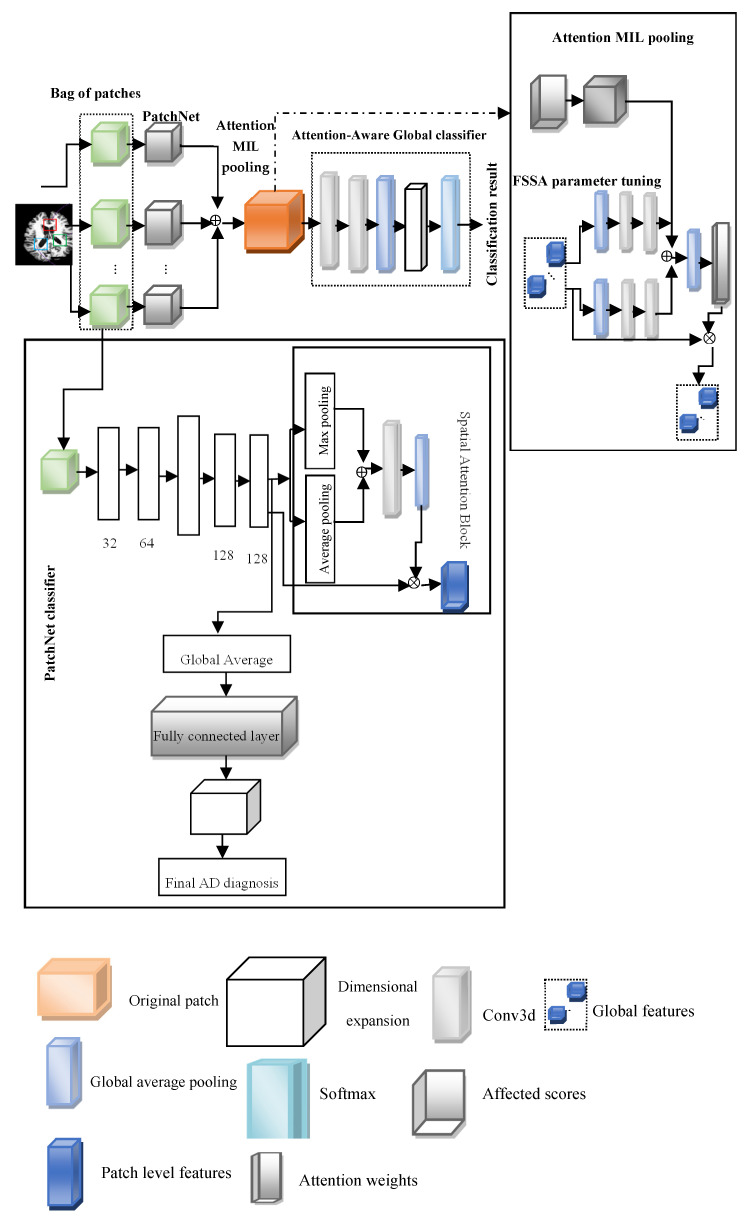
Fuzzy Optimized Attention Network with MIDL (FOA-MIDL).

This paper introduces the FOA-MIDL network to rapidly identify MCI and AD at the prodromal stage. To diagnose AD and determine discriminative pathological areas utilizing sMRI data, FOA-MIDL is implemented. Combining instance-level attributes into a global bag-level model of features is a crucial step in MIDL. As a multi-instance issue, the patch-level brain morphometric pattern is presented for the detection of AD and created utilizing MIL. The weight of every case is estimated using the attention method. The suggested method’s flow diagram is shown in Figure 1.

## 4. Image Preprocessing

The datasets used in this study were obtained from the AIBL (https://aibl.csiro.au) and the public ADNI database (http://adni.loni.usc.edu). The ADNI dataset consists of 1193 1.5T/3T T1-weighted sMRI scans obtained from participants during their initial screening visits, often known as the baseline, throughout three distinct ADNI stages: ADNI-1, ADNI-2, and ADNI-3. Given the clinical criteria, including the Clinical Dementia Rating (CDR) and scores from the Mini-Mental State Examination (MMSE), these patients can be classified as AD, MCI, and NC. To forecast MCI conversion, MCI participants are separated into two groups; stable MCI patients who maintained a diagnosis of MCI for 36 months following the baseline visit are denoted as sMCI. There are 389 AD, 232 sMCI, 172 pMCI, and 400 NC patients in the examined ADNI dataset. The AIBL dataset consists of baseline sMRI images from 496 distinct subjects, with 79 AD, 93 sMCI, 17 pMCI, and 307 NC patients. Table 1 displays the demographic information for these 1689 participants from the ADNI and AIBL databases.

To ensure generalizability, the model is trained and validated using data from the ADNI and AIBL datasets and then applied to domains with different imaging procedures and subject characteristics. The use of fuzzy attention mechanisms, which can handle uncertainty in anatomical variation, can improve domain-invariant feature extraction. Stability is quantified by stratified cross-validation across age, sex, and APOE4 genotype subgroups; saliency-based visualization, like Grad-CAM, verifies that therapeutically significant brain areas, like the entorhinal cortex and hippocampus, are consistently focused across cohorts.

Preprocessing is performed on the initial structured MRI data downloaded from the ADNI to improve feature learning and categorization. Initially, the original images in the 3D Neuroimaging Informatics Technology Initiative (NIfTI) format are normalized by employing 3D gradwarp adjustment for gradient nonlinearity and B1 nonuniformity correction for the intensity correction of nonuniformity. After this, the Colin27 template is subjected to linear registration to eliminate global linear variances and skull stripping on all sMRI images accordingly. This is achieved by employing the FSL toolbox’s “bet” instruction with a default fractional intensity threshold of 0.5 and the “flirt” instruction with a default setting of 12 DOF and correlation ratios as cost functions. Once they have been normalized to the Colin27 standard space, the size of the MRI images is 181 × 217 × 181 voxels.

## 5. Multi-Instance Learning (MIL)

When developing the suggested framework, we considered the patch-level brain morphometric form evaluation as a multi-instance issue that depends on multi-instance learning. *N* is the quantity of bags, $X_{i}$ is the *i*th bag, and $Y_{i}$ is the bag-level label of $X_{i}$. Many unlabeled occurrences are present in each bag. $X_{i}={\left\{I_{i,j}\right\}}_{j=1}^{N_{i}}$, where $I_{i,j}$ is the *j*th instance, and $N_{i}$ is the quantity of instances in $X_{i}$. All cases in a negative P bag are negative, but there is at least one positive case in a positive bag. Only when $\sum_{j=0}^{N_{i}}y_{i,j}$ is present, $Y_{i}=0$; otherwise, $Y_{i}=1$, where $y_{i,j}$ is the instance-level label of $I_{i,j}$. The following is the expression for the probability Θ of the positive class:

(1) Θ(X)=gϕf(X)

The four main components in the FOA-MIDL system are as follows: a set of transformed instances (A-MIDL), a transform f  for instance-level features (PatchNet), the choice of cases to compose a bag *X* (patch location proposal), and a classification *g* centered on the mixed bag-level feature (attention-aware global classifier) (see Figure 1).

## 6. Patch Location Proposal

We provide an original strategy incorporating a group evaluation on patch-level structures rather than voxel-wise attributes, drawing inspiration from patch extraction. To facilitate analyses and prevent unneeded data, we uniformly partition the MRI image into numerous cubic patches of a constant size (W × W × W) without overlapping. However, not every partitioned patch is related to the abnormalities brought about by AD. The significance of the disparities between the experimental and control groups is assessed using a *t*-test. Equation (2) is used to normalize each *p*-value that is determined for each location:

(2) $$\frac{pvalue-MIN}{MAX-MIN}$$

We create a *p*-value map that encompasses the entire brain MRI scan. Moreover, it is generally accepted that sites with smaller *p*-values exhibit greater discrimination. To create a bag, we choose several patches in a single image in the areas where the *p*-values are the lowest, based on the *p*-value map ($X=\{I_{1},\;I_{2},\ldots ,\;I_{k}\}$, where $I_{i}\in R^{W\times W\times W}$ and *k* is the number of selected patches input into the proposed system.

## 7. PatchNets with Spatial Attention Blocks

PatchNet involves (1) acquiring knowledge of a spatially aware patch-level feature model and (2) producing an affect score that signifies the capacity to activate the bag label. In fixed-size patches, spatial attention blocks are utilized to improve the features of discriminative sections.

### 7.1. PatchNet

The primary component of PatchNet is the former, which tries to minimize the size of the feature maps while acquiring more abstract representations of features from the original patches. Four 3D convolutional layers constitute this structure, with a max pooling layer in the center to adjust the size of the input patches. Rectified linear unit (ReLU) activation and batch normalization (BN) occur after each convolutional layer. PatchNet forms two branching sections according to the feature maps produced by Conv4.

### 7.2. Spatial Attention Blocks

The spatial attention component creates the spatial attention block, which is intended to be implanted into PatchNet to modify the local extracted features from the 3D image patch [29]. Two distinct pooling techniques—channel average pooling and channel max pooling—are used to create two feature maps with the names of the maximum and average features. Next, as the input of the next convolution layer (stride: 1, kernel size: 3×3×3, padding: 1 to preserve the feature map size), the two feature maps are concatenated with a size of 2×w×w×w. One way to conceptualize the convolutional layer’s output is to use spatial attention maps ($A_{spatial}\in R^{\;w\times w\times w})$, with the same size as the convolutional layer feature maps, whereby each location’s attention score is constrained by the sigmoid layer to a value between 0 and 1. The spatial attention map indicates which portion of a patch to highlight or repress in the feature representation by describing the spatially varying inputs from various regions within a patch. The computed attention map $A_{spatial}$ is multiplied element-wise by each feature map in the Conv4 output to produce the final local spatial attention-aware structure model. Let $F=\left\{F_{1},F_{2},\ldots .,F_{C}\right\}$ represent the output of Conv4, where $F_{i}\in R^{\;w\times w\times w}$ and *C* is the number of channels. The expression for max pooling along the channel axis is as follows:

(3) $$F_{max}=ChannelMaxPooling(F)$$

where $F_{max}^{w,h,l}=max\{F_{1}^{w,h,l},F_{2}^{w,h,l},\ldots .,F_{C}^{w,h,l}\}$. Across the channel axis, average pooling is indicated as

(4) $$F_{average}=ChannelAveragePooling(F)$$

where $F_{average}^{w,h,l}=\frac{1}{C}\sum_{c=1}^{C}F_{c}^{w,h,l}$. Next, we create a spatial attention map by concatenating the two feature maps:

(5) $$A_{spatial}=\sigma (W[F_{max};F_{average}])$$

(6) $$F=\left[F_{1}⊗A_{spatial};\ldots ;F_{C}⊗A_{spatial}\right]$$

where ⊗  is element-wise multiplication.

## 8. Attention MIL Pooling

To learn a patch attention map that shows the comparative involvement of every patch, the A-MIDL technique is developed. The initial step in compressing each patch-level structural form ($F\in R^{\;C\times w\times w\times w})$ generated from PatchNet is average pooling across the channel axis to $\bar{F}\in R^{\;1\times w\times w\times w}$. Subsequently, $F_{global}=\left\{{\bar{F}}_{1},\;{\bar{F}}_{2},\ldots ,\;{\bar{F}}_{c}\right\}$, where C denotes the number of patches and ${\bar{F}}_{i}$ denotes the patch-level features of the ith input patch. This is created by concatenating the compressed patch-level characteristics with the global feature representation. Due to empirical evidence showing that employing these feature descriptors can increase a network’s representation power more than utilizing just one, global max pooling (GMP) and global average pooling (GAP) are applied in conjunction to generate two distinct feature descriptors. After this, two 1 × 1 × 1 convolutional layers are matched to the two descriptors to learn two more patch attention maps:

(7) $$A_{average}=W_{1}b_{1}ReLU\left(W_{0}b_{0}GAP\left(F_{average}\right)\right)$$

(8) $$A_{max}=W_{1}b_{1}ReLU\left(W_{0}b_{0}GMP\left(F_{global}\right)\right)$$

$W_{0}$, $W_{1}$ are the weights of the convolutional layers; $b_{0},\;b_{1}$ represent the bias of the classifier. The convolution layers analyze the average feature descriptors and the maximum feature descriptor, which is shared. To assess the effect of each patch, the effect score learned from every intra-patch feature is taken into consideration, in addition to the two patch attention maps learned from inter-patch connections. An impact vector $a=\{a_{1},a_{2},\ldots ,a_{C}\}$ is formed by the effect scores of each PatchNet, where *C* is the total number of patches. The sizes of the effect vectors and the patch attention maps are the same. Therefore, by combining the three distinct attention maps, comprehensive patch attention maps $A_{patch}$ can be created by element-wise summing. The sigmoid function σ then activates the patch attention map:

(9) $$A_{patch}=\sigma (A_{average}+A_{max}+a)$$

Ultimately, the attention-aware global feature model is created by multiplying the prior global models with the patch attention map $F_{global}$:

(10) $$F_{global}=F_{global}⊗A_{patch}$$

where ⊗ represents tensor multiplication. A-MIDL may decrease the misdiagnosis rate of specific subjects while enhancing the accuracy in classification by emphasizing certain features for critical patches. Thus, it reduces noise interference and maintains the connections among critical and unimportant patches to prevent the loss of potentially relevant attributes. In particular, problematic sites can be identified using the patch attention map as a reference.

### 8.1. Fuzzy Salp Swarm Algorithm (FSSA)

The FSSA is an environment-inspired optimization technique used for parameter optimization in MIL [30,31]. The salp X population is composed of N agents with d-dimensions. It is described by an N×d-dimensional parameter matrix, defined in Equation (11) [32]:

(11) $$X_{i}=\lfloor x_{1}^{1}x_{2}^{1}\ldots x_{d}^{1}x_{1}^{2}x_{2}^{.2}\ldots x_{d}^{2}\;\vdots \;\vdots \ldots \;\vdots \;\;x_{1}^{N}x_{2}^{N}\ldots x_{d}^{N}\;\;\rfloor$$

Equation (12) is employed in the SSA to determine the leader’s position:

(12) $$X_{j}^{1}=\left\{\begin{array}{cc} F_{j}+C_{1}\left(\left({ub}_{j}-{lb}_{j}\right)\right)C_{2}+{lb}_{j}, & C_{3}\geq 0.5\\ F_{j}-C_{1}\left(\left({ub}_{j}-{lb}_{j}\right)\right)C_{2}+{lb}_{j}), & C_{3}<0.5 \end{array}\right.$$

where $X_{j}^{1}$ signifies the leader’s position, $F_{j}$ denotes the parameter location vector of the food source in the *j*th dimension, ${ub}_{j}$ is the upper limit of the *j*th parameter dimension, and ${lb}_{j}$ represents the lower limit of the *j*th parameter dimension. $C_{2}$ and $C_{3}$ are random values within [0, 1], being the key parameters of the system, as demonstrated in Equation (13):

(13) $$C_{1}=2e^{-{\left(T_{max}\right)}^{2}}$$

where t is the iteration, while $T_{max}$ denotes the maximum number of iterations. This variable becomes smaller as the quantity of iterations increases. The location of followers is modified by Equation (14):

(14) $$X_{j}^{i}=\frac{X_{j}^{i}+X_{j}^{i-1}}{2}$$

where i≥2  and $x_{j}^{i}$ is the location of the *i*th follower salp at the *j*th parameter. The FSSA begins by randomly initializing a classifier variable within a particular range. Equation (15) is used to express this:

(15) $$x_{ij}=x_{min.j}+U\left(0,1\right)\times \left(x_{min.j}-x_{max.j}\right)$$

where i lies in the range [1, 2, 3, …, *n*], j is [1, 2, 3, …, *D*], $x_{ij}$ is the *i*th image for the *j*th parameter, $x_{min.j}$ and $x_{max.j}$ are the lower and upper bounds of the parameters in the case of AD, and $U\left(0,1\right)$ is a uniform random number in the range [0, 1]. Salps are planktonic tunicates with a barrel form that swarm in the water in a unique chain pattern. A leader–follower dynamic controls their movement; the first salp sets the course for the swarm, and the subsequent salps change their positions depending on the leader and their predecessor. In the FSSA, the computational model of this biological process involves splitting the population into leaders who are responsible for exploring the whole area and followers who are responsible for exploiting resources close to home by adjusting their relative positions according to those of the leaders. Convergence toward optimum solutions may be smooth and adaptable thanks to the chain structure. When applied to the FSSA, fuzzy logic models the uncertainty inherent in salps’ sensory input and movement choices, making the model more biologically plausible. For feature spaces created from high-dimensional or noisy data, like sMRI data, fuzziness enables each agent to perform probabilistic rather than deterministic updates, which enables improved convergence.

### 8.2. Position Updating

The SSA algorithm includes exploration and exploitation procedures. The two processes of exploration and exploitation determine a system’s level of functionality. Thus, the method is improved by dividing the iterations into two halves; novel equations are employed for the first half, and the approach is employed for the second half (where much exploitation is needed). The newly updated equations are extended by Equations (16) and (17):

(16) $$x_{1}=x_{i}-A_{1}\left(C_{1}x_{new}-x_{i}^{t}\right);x_{2}=x_{i}-A_{2}(C_{2}x_{new}-x_{i}^{t})\;x_{3}=x_{i}-A_{3}(C_{3}x_{new}-x_{i}^{t})$$

(17) $$x_{new}^{t+1}=\frac{x_{1}+x_{2}+x_{3}}{3}$$

(18) $$x_{new}^{t+1}=x_{new}^{t}+\alpha \left(x_{best}-x_{new}^{t}\right)$$

where $x_{new}$ is the newly optimized MIL parameter from the randomized populace within the search space for any disease diagnosis, and $A_{1},A_{2},A_{3}$ and $C_{1},C_{2},C_{3}$ are derived from $A=2ar_{1}-a$ and $C_{2}.\;\;r_{2}$ and *a* decrease linearly within the range [0, 2]. α is generated using the Gaussian fuzzy membership (GFM) function. The GFM function is denoted as Gaussian(x:c,s), where *s*, *c* denote the standard deviation and mean, given by Equation (19):

(19) $$\mu_{A}(x,c,s,m)=exp\left[-\frac{1}{2}{\left|\frac{x-c}{s}\right|}^{m}\right]$$

### 8.3. Selection Operation

The suggested system uses greedy selection, which checks if the newly created parameters are superior to the previous optimal variable. $F\left(x_{i}^{t}\right)$ indicates the fitness for the $x_{i}^{t}$ solution, and the selection process is shown in Equation (20):

(20) $$x_{new}^{t+1}=\{x_{new}x_{i}^{t}{if}_{otherwise}^{F\left(x_{new}\right)<F(x_{i}^{t})}$$

### 8.4. Controlling Parameters

The controlling parameter $c_{1}$ defines this stage. The lower $c_{min}$ and upper $c_{max}values$ for the controlling variable $c_{1}$ are within [0.05, 0.95], and the general adaptive formula is shown in Equation (21):

(21) $$c_{1}=c_{max}+\left(c_{min}-c_{max}\right)\times \left(a+\frac{10t}{T_{max}}\right)$$

where *c* is the inertia weight parameter and a∈[0,1] is any random number; t and $T_{max}$ are the current and maximum numbers of iterations.

### 8.5. Population Adaptation

The run time required to obtain nearly optimal solutions for parameters like weight and bias is called the computational complexity. The overall computational complexity is proportional to the population size n, dimension size d, and maximum number of function evaluations $T_{max}$, and it is specified by $O(n.d.T_{max})$. The population adaption process is shown in Equation (22):

(22) $$n\left(g+1\right)=round\left[\left(\frac{n_{min-}n_{max}}{T_{max}}\right).T_{max}+n_{max}\right]$$

where $n_{max}\;and\;n_{min}$ denote the maximum and minimum population sizes, and $T_{max}$ is the maximum number of iterations. The pseudocode for the FSSA is shown in Algorithm 1.
**Algorithm 1**. Fuzzy Salp Swarm Algorithm (FSSA) INPUT: Structural MRI data from ADNI and AIBL datasetsOBJECTIVE FUNCTION: Classifier accuracy, $f\left(x_{i}\right)$, accuracy of the MIL classifierOUTPUT: Optimized parameters (weights and biases) of the MIL classifier            1.Begin            1.1Establish a starting populace of n butterflies $x_{i}=(i=1to\;n)$, $c_{1}$, via the number of parameters in the classifier            1.2Evaluate the fitness $f\left(x_{i}\right)$ for each salp            1.3Appraise and choose the best salp            2.Until termination criteria are met            3.If iterations <MI/2            3.1Discover a novel solution employing Equation (18)            3.2Estimate fitness $f\left(x_{i}\right)$            3.3Modify $c_{1}$ utilizing Equation (21) and *n* by Equation (22)            4.Else            4.1Find $x_{new}$ new solution by Equation (18)            4.2Estimate fitness $f\left(x_{i}\right)$ for each salp            4.3Update $c_{1}$ by Equation (21) and *n* by Equation (22)               5.End                        Obtain the best-optimized parameters like weight and bias            6.End until                        Update the final best range of weight and bias of the classifier            7.End

## 9. Attention-Aware Global Classifier

Strong correlations between patches are taken into consideration by the attention-aware global classifier as it proceeds with analyzing the bag-level representations $F_{global}$. A two-layer convolutional network is placed before the global classifier to extract additional structural data from the MIL pooling and squeeze the feature maps across channels. This network is utilized to generate the attention-aware feature model. Attention-aware global classifiers are intended to acquire the essential features for the entire set of brain data and provide categorization results while estimating MCI progression. The outcome depends on the various weighted feature map results from the prior A-MIDL.

## 10. Loss Function

While patch-level labels are unclear, image-level labels are provided; this makes the image-level label the only guide utilized in backpropagation to update the network weights W. According to the cross-entropy loss, the loss function utilized to train the framework is

(23) $$L\left(W\right)=-\frac{1}{N}\sum_{n=1}^{N}\log\left(P\left(Y_{n}|X_{n};W\right)\right)$$

where *N* is the number of images, and $P\left(Y_{n}|X_{n};W\right)\;$ is the probability that $X_{n}$ will correctly predict the outcome. In a complete network, the global classifier backpropagates the training losses to the PatchNet and MIL pooling, which assist in updating the network’s settings utilizing an optimization technique. The network eventually learns a map by minimizing the loss function from *X* to *Y*.

## 11. Experiments and Discussion

The efficacy and generalizability of the disease identification technique are assessed utilizing the AIBL and ADNI data. We separate the ADNI data into training and test datasets, with 80% of the samples being utilized for model training and the other 20% serving as a hold-out dataset. The hyperparameters are selected, and the algorithm is trained to utilize a five-fold cross-validation approach that employs the ADNI training dataset. Next, the held-out ADNI test dataset is employed to evaluate the trained algorithm with improved hyperparameters. This is also performed on the AIBL dataset. The disease diagnosis method is verified on a variety of AD diagnostic tasks, including MCI classification (pMCI vs. NC and sMCI vs. NC), AD categorization (AD vs. NC), and MCI conversion prediction (pMCI vs. sMCI).

## 12. Evaluation Metrics

Four metrics, namely the accuracy (ACC), SPE, SEN, and the area under the receiver operating characteristic curve (AUC), are used to evaluate the classification efficiency. These metrics are shown below:

(24) $$ACC=\frac{TP+TN}{TP+TN+FP+FN}$$

(25) $$SEN=\frac{TP}{TP+FN}$$

(26) $$SPE=\frac{TN}{TN+FP}$$

Here, true negatives, true positives, false positives, and false negatives are denoted by TN, TP, FP, and FN. The AUC is computed by varying the thresholds applied to the prediction outcomes for every feasible pair of true positive rates (TPR = SEN) and false positive rates (FPR = 1 − SPE).

## 13. Competing Methods

The FOA-MIDL method is compared with three modern patch-level DL approaches (landmark deep MIL (LDMIL) [33], hierarchical fully convolutional network (HFCN) [34], and dual attention MIDL (DA-MIDL)). Table 2 displays the results regarding the FOA-MIDL framework’s efficiency in AD categorization and MCI conversion forecasting compared to that of the other techniques on the ADNI test set. Additionally, the five-fold validation results of this approach using the ADNI training set are presented. Table 2 illustrates that, in most situations, the FOA-MIDL framework performs better in AD categorization and MCI conversion prediction. Regarding AD categorization, the FOA-MIDL approach performs better across all four metrics (ACC = 0.938, SPE = 0.949, SEN = 0.936, and AUC = 0.973).

As shown in Table 3, the FOA-MIDL system performs better in the two classification tests. In differentiating pMCI patients from normal controls in a classification task, for instance, the FOA-MIDL system yields superior outcomes (ACC = 0.895, SPE = 0.925, SEN = 0.824, and AUC = 0.917). The FOA-MIDL approach also yields much greater accuracy in categorizing sMCI cases and normal controls, particularly in terms of the AUC (0.875) and ACC (0.825).

We employ an independent AIBL database to assess the FOA-MIDL system and its rival techniques trained on the ADNI database to confirm the generalizability of the FOA-MIDL system. Table 4 displays the AD categorization and MCI conversion forecasting results when utilizing the AIBL database.

FOA-MIDL achieves the best results (i.e., ACC = 0.927, SEN = 0.876, SPE = 0.952, and AUC = 0.965) for the AD vs. NC categorization task on the AIBL database. The FOA-MIDL system outperforms the second-best approach (0.816, 0.835, 0.709, and 0.827 for ACC, SPE, SEN, and AUC, respectively) in the pMCI vs. sMCI classification test, yielding greater outcomes (0.837, 0.848, 0.735, and 0.839 for SPE, ACC, AUC, and SEN, respectively). The FOA-MIDL approach works well on a variety of datasets.

## 14. Comparison with Previous Works

We compare the suggested approach with its equivalents—namely a framework with no attention module (N-MIDL), a simulation only with a spatial attention block (S-MIDL), a simulation only with A-MIDL, and DA-MIDL—to assess the efficacy of the attention components utilized in this research. We test these four approaches on two tasks linked to AD detection and present the findings in Table 5. The suggested attention system can generally enhance the categorization accuracy, as Table 5 illustrates.

Table 6 lists various recent findings presented in similar research employing sMRI data from the ADNI dataset in AD categorization and MCI conversion forecasting tasks, enabling an exhaustive comparison between the suggested approach and associated research on the efficacy of AD evaluation, involving ROI-level features (multi-kernel+kNN) [35] and patch-level features (K-Means+DenseNet [19], Attention+MIL+CNN [26], and Attention+FOA-MIDL+CNN).

**Figure 2:**
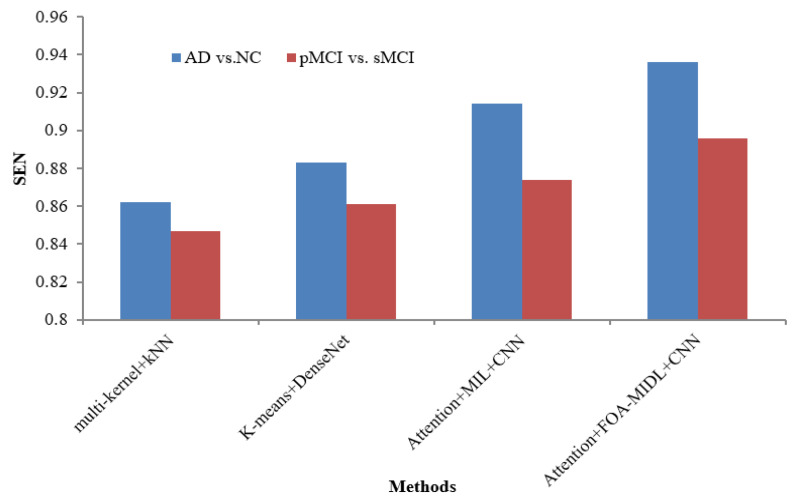
SEN comparison of sMRI-based studies.

**Figure 3:**
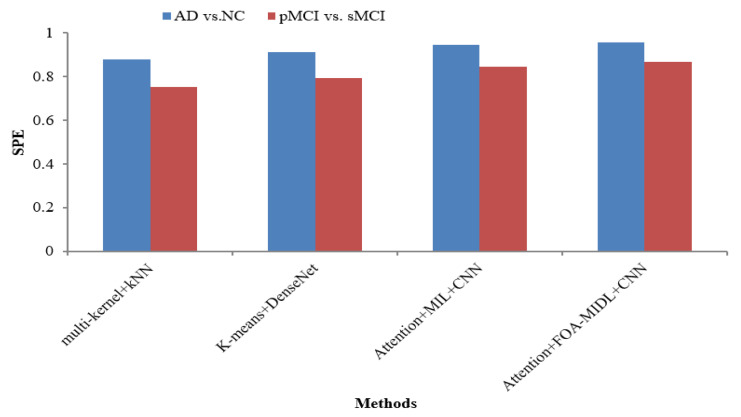
SPE comparison of sMRI-based studies.

Figure 2 shows the performance comparison in terms of the SEN metric concerning sMRI-based studies (multi-kernel+kNN, K-Means+DenseNet, Attention+MIL+CNN, and Attention+FOA-MIDL+CNN) across two different datasets. The results show that the suggested classifier has the highest SEN outcomes of 89.60% and 93.60% for pMCI vs. sMCI and AD vs. NC. The other methods, namely multi-kernel+kNN, K-Means+DenseNet, and Attention+MIL+CNN, give the lowest SEN values of 86.20%, 88.30%, and 91.40% for the AD vs. NC dataset (refer to Table 6). The SPE metric comparison concerning sMRI-based studies (multi-kernel+kNN, K-Means+DenseNet, Attention+MIL+CNN, and Attention+FOA-MIDL+CNN) regarding two dissimilar datasets is illustrated in Figure 3. The results show that the proposed classifier has the highest SPE results of 86.60% and 95.60% for pMCI vs. sMCI and AD vs. NC. The other methods, namely multi-kernel+kNN, K-Means+DenseNet, and Attention+MIL+CNN, give the lowest SPE results of 87.70%, 91.20%, and 94.40% for the AD vs. NC dataset (refer to Table 6).

**Figure 4:**
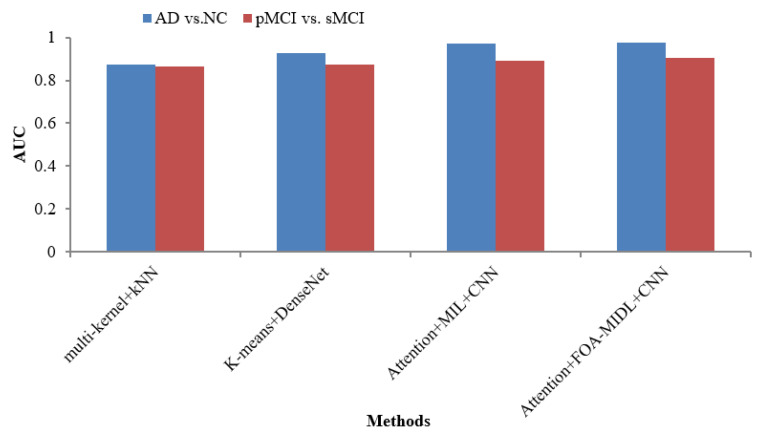
AUC comparison of sMRI-based studies.

The AUC metric comparison concerning sMRI-based studies (multi-kernel+kNN, K-Means+DenseNet, Attention+MIL+CNN, and Attention+FOA-MIDL+CNN) regarding two dissimilar datasets is illustrated in Figure 4. The outcomes show that the suggested classifier has the maximum AUC results of 90.30% and 97.50% for pMCI vs. sMCI and AD vs. NC. The other methods, namely multi-kernel+kNN, K-Means+DenseNet, and Attention+MIL+CNN, give the lowest AUC scores of 87.20%, 92.70%, and 96.90% for the AD vs. NC dataset (refer to Table 6).

**Figure 5:**
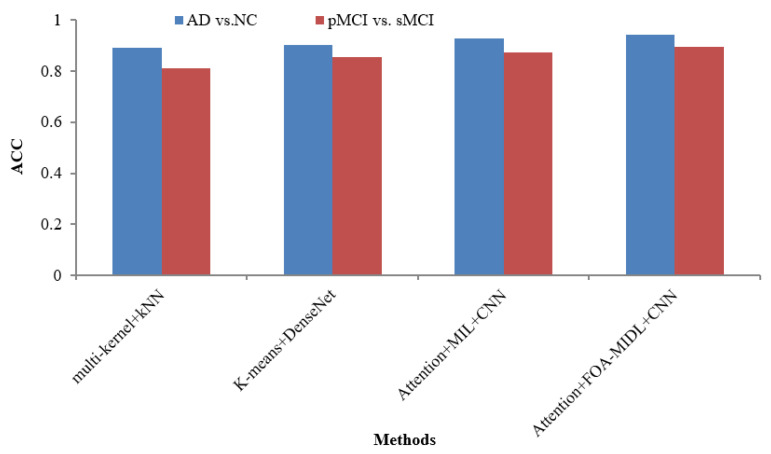
ACC comparison of sMRI-based studies.

The AUC metric comparison concerning sMRI-based studies (multi-kernel+kNN, K-Means+DenseNet, Attention+MIL+CNN, and Attention+FOA-MIDL+CNN) regarding two dissimilar datasets is illustrated in Figure 4. In Figure 5, the outcomes show that the suggested classifier has the maximum ACC results of 89.50% and 94.30% for pMCI vs. sMCI and AD vs. NC. The other methods, namely multi-kernel+kNN, K-Means+DenseNet, and Attention+MIL+CNN, give the lowest ACC scores of 89.30%, 90.40%, and 92.80% for the AD vs. NC dataset (refer to Table 6).

The proposed FOA-MIDL model enhances clinical decision making by generating attention-based heatmaps directly highlighting discriminative brain regions, such as the hippocampus and entorhinal cortex, from sMRI inputs. The model adds Grad-CAM and patch-level attention visualization to provide spatial interpretability so that clinicians can understand which anatomical areas affect the classification outcome. This increases the confidence in model predictions and supports alignment with known neuropathological patterns of AD.

## 15. Ablation Study

We carried out a full ablation study to determine the contribution of each module in the FOA-MIDL architecture by eliminating and altering specific parts. These parts included the CNN-based PatchNet, the attention mechanism (A-MIDL), and the fuzzy salp swarm algorithm (FOA optimization). The findings (see Table 7) show that removing the CNN-based PatchNet exerted the greatest effect on the classification accuracy and AUC, showing its importance in extracting spatial features. The elimination of the attention module additionally caused a decrease, which shows its importance in improving the patch-level contributions to global diagnosis. Additionally, eliminating the FOA optimization marginally decreased the overall accuracy and AUC, validating its efficacy in calibrating the classifier’s parameters for optimal outcomes. These results show that all three parts interact in order to improve the diagnostic accuracy, indicating that their inclusion in the suggested design is warranted.

## 16. Conclusions and Future Work

A novel FOA-MIDL system is introduced for sMRI analysis and Alzheimer’s detection. It includes the following components: (1) a PatchNet with spatial attention blocks, which optimizes the features of aberrantly altered microstructures in the brain while extracting discriminative structures inside each sMRI patch; (2) an attention-aware global classifier to provide decisions about the classification of AD according to the integrated feature image for the full brain; (3) an A-MIDL procedure to balance the relative contributions of every patch; (4) the fuzzy salp swarm algorithm (FSSA), which optimizes the parameters of the global classifier to resolve the problem of restricted samples and raise the efficiency of the framework. The FSSA is an environment-inspired parameter optimization tool with a Gaussian fuzzy membership function. Utilizing the various weighted feature maps optimized by the FSSA to produce outcomes for AD categorization, the attention-aware global classifier is intended to train integral feature representations for sMRI data. A total of 1689 patients’ data from two datasets (ADNI and AIBL) are used to assess the FOA-MIDL system in several AD-related diagnoses. Utilizing measures like the SEN, SPE, AUC, and ACC, the FOA-MIDL system is contrasted with the most advanced approaches. A variety of AD diagnostic tasks, encompassing AD vs. NC, pMCI vs. sMCI, pMCI vs. NC, and sMCI vs. NC, are used to validate the classifier. We summarize the remaining problems as follows: (1) the input patch sizes are uniform and fixed; (2) the group comparison-based patch location suggestions are separated from the next network. This indicates that the suggested approach is not a complete analytical process, which could impact the framework’s effectiveness.

## Figures and Tables

**Table 1:** Demographic details.

Dataset	Group Type	Gender (Male/Female)	Age (Mean ± Std)	MMSE (Mean ± Std)	CDR (Mean ± Std)
ADNI	AD	202/187	75.13 ± 7.86	23.28 ± 2.03	0.75 ± 0.25
	pMCI	105/67	75.73 ± 7.05	26.59 ± 1.71	0.50 ± 0.00
	sMCI	155/77	76.40 ± 7.94	27.27 ± 1.78	0.49 ± 0.04
	NC	202/198	73.85 ± 6.38	29.10 ± 1.01	0.00 ± 0.00
AIBL	AD	33/46	73.34 ± 7.77	20.42 ± 5.46	0.95 ± 0.51
	pMCI	9/8	75.29 ± 6.16	26.24 ± 2.04	0.47 ± 0.13
	sMCI	48/45	74.67 ± 7.21	27.23 ± 2.08	0.46 ± 0.12
	NC	134/173	73.12 ± 6.19	28.77 ± 1.25	0.02 ± 0.20

**Table 2:** Outcomes for AD classification and MCI conversion forecasting on ADNI test set.

Dataset	Method	SEN	SPE	AUC	ACC
AD vs. NC	LDMIL	0.864	0.928	0.954	0.896
	HFCN	0.898	0.914	0.945	0.908
	DA-MIDL	0.915	0.939	0.967	0.926
	FOA-MIDL	0.936	0.949	0.973	0.938
pMCI vs. sMCI	LDMIL	0.718	0.808	0.795	0.769
	HFCN	0.687	0.855	0.824	0.785
	DA-MIDL	0.779	0.832	0.857	0.815
	FOA-MIDL	0.835	0.867	0.881	0.854

**Table 3:** Outcomes for pMCI vs. NC and sMCI vs. NC classification on ADNI test set.

Dataset	Method	SEN	SPE	AUC	ACC
pMCI vs. NC	LDMIL	0.737	0.929	0.912	0.875
	HFCN	0.798	0.916	0.920	0.887
	DA-MIDL	0.829	0.926	0.919	0.898
	FOA-MIDL	0.856	0.942	0.938	0.916
sMCI vs. NC	LDMIL	0.788	0.807	0.817	0.796
	HFCN	0.725	0.858	0.838	0.813
	DA-MIDL	0.816	0.844	0.867	0.829
	FOA-MIDL	0.825	0.856	0.875	0.842

**Table 4:** Outcomes for AD classification and MCI conversion forecasting on AIBL dataset.

Dataset	Method	SEN	SPE	AUC	ACC
AD vs. NC	LDMIL	0.778	0.896	0.905	0.871
	HFCN	0.827	0.908	0.938	0.893
	DA-MIDL	0.851	0.924	0.946	0.908
	FOA-MIDL	0.876	0.952	0.965	0.927
pMCI vs. sMCI	LDMIL	0.615	0.803	0.798	0.772
	HFCN	0.653	0.816	0.802	0.788
	DA-MIDL	0.709	0.835	0.827	0.816
	FOA-MIDL	0.735	0.848	0.839	0.837

**Table 5:** Outcomes for AD classification and MCI conversion forecasting achieved by methods on ADNI test set.

Dataset	Method	SEN	SPE	AUC	ACC
AD vs. NC	N-DMIL	0.889	0.894	0.951	0.895
	S-DMIL	0.902	0.905	0.956	0.907
	A-DMIL	0.909	0.918	0.965	0.916
	DA-MIDL	0.916	0.943	0.971	0.938
	FOA-MIDL	0.926	0.955	0.976	0.946
pMCI vs. sMCI	N-DMIL	0.748	0.772	0.765	0.757
	S-DMIL	0.721	0.807	0.794	0.771
	A-DMIL	0.805	0.769	0.813	0.786
	DA-MIDL	0.788	0.837	0.856	0.815
	FOA-MIDL	0.856	0.862	0.871	0.847

**Table 6:** sMRI comparison across studies for AD and MCI.

Dataset	Method	SEN	SPE	AUC	ACC
AD vs. NC	Multi-Kernel+kNN	0.862	0.877	0.872	0.893
	K-Means+DenseNet	0.883	0.912	0.927	0.904
	Attention+MIL+CNN	0.914	0.944	0.969	0.928
	Attention+FOA-MIDL+CNN	0.936	0.956	0.975	0.943
pMCI vs. sMCI	Multi-Kernel+kNN	0.847	0.753	0.866	0.812
	K-Means+DenseNet	0.861	0.792	0.874	0.856
	Attention+MIL+CNN	0.874	0.845	0.889	0.873
	Attention+FOA-MIDL+CNN	0.896	0.866	0.903	0.895

**Table 7:** Performance comparison via ablation study (AD vs. NC classification on ADNI).

Model Variant	Accuracy (%)	AUC (%)	Sensitivity (%)	Specificity (%)
Full FOA-MIDL (Proposed)	94.6	97.6	92.6	95.5
Without CNN (PatchNet)	87.5	91.1	88.9	89.4
Without Attention Mechanism	89.5	93.2	90.9	91.8
Without FOA Optimization	90.3	94.5	91.1	92

## Data Availability

The datasets adopted in the current study are publicly available online.

## References

[1] Bayram E., Caldwell J.Z., Banks S.J. (2018). Current understanding of magnetic resonance imaging biomarkers and memory in Alzheimer’s disease. Alzheimer’s Dement. Transl. Res. Clin. Interevent..

[2] Shi J., Zheng X., Li Y., Zhang Q., Ying S. (2018). Multimodal neuroimaging feature learning with multimodal stacked deep polynomial networks for diagnosis of Alzheimer’s disease. IEEE J. Biomed. Health Inform..

[3] Leandrou S., SPetroudi PAKyriacou C., Reyes-Aldasoro C., Pattichis C.S. (2018). Quantitative MRI brain studies in mild cognitive impairment and Alzheimer’s disease: A methodological review. IEEE Rev. Biomed. Eng..

[4] Vounou M., Janousova E., Wolz R., Stein J.L., Thompson P.M., Rueckert D., Montana G., Alzheimer’s Disease Neuroimaging Initiative (2012). Sparse reduced-rank regression detects genetic associations with voxel-wise longitudinal phenotypes in Alzheimer’s disease. Neuroimage.

[5] Shao W., Peng Y., Zu C., Wang M., Zhang D., Alzheimer’s Disease Neuroimaging Initiative (2020). Hypergraph based multi-task feature selection for multimodal classification of Alzheimer’s disease. Comput. Med. Imaging Graph..

[6] Qiu S., Joshi P.S., Miller M.I., Xue C., Zhou X., Karjadi C., Chang G.H., Joshi A.S., Dwyer B., Zhu S. (2020). Development and validation of an interpretable deep learning framework for Alzheimer’s disease classification. Brain.

[7] Martí-Juan G., Sanroma-Guell G., Piella G. (2020). A survey on machine and statistical learning for longitudinal analysis of neuroimaging data in Alzheimer’s disease. Comput. Methods Programs Biomed..

[8] Tanveer M., Richhariya B., Khan R.U., Rashid A.H., Khanna P., Prasad M., Lin C.T. (2020). Machine learning techniques for the diagnosis of Alzheimer’s disease: A review. ACM Trans. Multimed. Comput. Commun. Appl..

[9] Wu E.Q., Hu D., Deng P.-Y., Tang Z., Cao Y., Zhang W.-M., Zhu L.-M., Ren H. (2021). Nonparametric bayesian prior inducing deep network for automatic detection of cognitive status. IEEE Trans. Cybern..

[10] Zhang J., Gao Y., Gao Y., Munsell B.C., Shen D. (2016). Detecting anatomical landmarks for fast alzheimer’s disease diagnosis. IEEE Trans. Med. Imaging.

[11] AbdulAzeem Y.M., Bahgat W.M., Badawy M.M. (2021). A CNN based framework for classification of Alzheimer’s disease. Neural Comput. Appl..

[12] Qiao H., Chen L., Ye Z., Zhu F. (2021). Early Alzheimer’s disease diagnosis with the contrastive loss using paired structural MRIs. Comput. Methods Prog. Biomed..

[13] Carbonneau M.-A., Cheplygina V., Granger E., Gagnon G. (2018). Multiple instance learning: A survey of problem characteristics and applications. Pattern Recogn..

[14] Dimitriou N., Arandjelović O., Caie P.D. (2019). Deep learning for whole slide image analysis: An overview. Front. Med..

[15] Ilse M., Tomczak J., Welling M. Attention-based deep multiple instance learning. Proceedings of the 35th International Conference on Machine Learning.

[16] Yao J., Zhu X., Jonnagaddala J., Hawkins N., Huang J. (2020). Whole slide images-based cancer survival prediction using attention guided deep multiple instance learning networks. Med. Image Anal..

[17] Yao Q., Wang R., Fan X., Liu J., Li Y. (2020). Multi-class arrhythmia detection from 12-lead varied-length ECG using attention-based time-incremental convolutional neural network. Inf. Fusion..

[18] Lama R.K., Gwak J., Park J.S., Lee S.W. (2017). Diagnosis of Alzheimer’s disease based on structural MRI images using a regularized extreme learning machine and PCA features. J. Healthc. Eng..

[19] Li F., Liu M. (2018). Alzheimer’s disease diagnosis based on multiple cluster dense convolutional networks. Comput. Med. Imaging Graph..

[20] Cui R., Liu M. (2019). Hippocampus analysis by combination of 3-D densenet and shapes for Alzheimer’s disease diagnosis. IEEE J. Biomed. Health Inform..

[21] Rallabandi V.S., Tulpule K., Gattu M. (2020). Automatic classification of cognitively normal, mild cognitive impairment and Alzheimer’s disease using structural MRI analysis. Inform. Med. Unlocked.

[22] Wang S., Wang H., Cheung A.C., Shen Y., Gan M. (2020). Ensemble of 3D densely connected convolutional network for diagnosis of mild cognitive impairment and Alzheimer’s disease. Deep. Learn. Appl..

[23] Zhao X., Ang C.K.E., Acharya U.R., Cheong K.H. (2021). Application of Artificial Intelligence techniques for the detection of Alzheimer’s disease using structural MRI images. Biocybern. Biomed. Eng..

[24] Chen Y., Xia Y. (2021). Iterative sparse and deep learning for accurate diagnosis of Alzheimer’s disease. Pattern Recognit..

[25] Feng J., Zhang S.W., Chen L., Xia J. (2021). Alzheimer’s disease classification using features extracted from nonsubsampled contourlet subband-based individual networks. Neurocomputing.

[26] Zhu W., Sun L., Huang J., Han L., Zhang D. (2021). Dual attention multi-instance deep learning for Alzheimer’s disease diagnosis with structural MRI. IEEE Trans. Med. Imaging.

[27] Uchida Y., Onda K., Hou Z., Troncoso J.C., Mori S., Oishi K. (2023). Microstructural Neurodegeneration of the entorhinal-Hippocampus pathway along the Alzheimer’s Disease Continuum. J. Alzheimer’s Dis..

[28] Uchida Y., Nishimaki K., Soldan A., Moghekar A., Albert M., Oishi K. (2024). Acceleration of brain atrophy and progression from normal cognition to mild cognitive impairment. JAMA Netw. Open.

[29] Woo S., Park J., Lee J.Y., Kweon I.S. CBAM: Convolutional block attention module. Proceedings of the European Conference on Computer Vision (ECCV).

[30] Abualigah L., Shehab M., Alshinwan M., Alabool H. (2020). Salp swarm algorithm: A comprehensive survey. Neural Comput. Appl..

[31] Faris H., Mirjalili S., Aljarah I., Mafarja M., Heidari A.A. (2020). Salp swarm algorithm: Theory, literature review, and application in extreme learning machines. Nature-Inspired Optimizers: Theories, Literature Reviews and Applications.

[32] Zhang H., Cai Z., Ye X., Wang M., Kuang F., Chen H., Li C., Li Y. (2022). A multi-strategy enhanced salp swarm algorithm for global optimization. Eng. Comput..

[33] Liu M., Zhang J., Adeli E., Shen D. (2018). Landmark-based deep multi-instance learning for brain disease diagnosis. Med. Image Anal..

[34] Lian C., Liu M., Zhang J., Shen D. (2020). Hierarchical fully convolutional network for joint atrophy localization and Alzheimer’s disease diagnosis using structural MRI. IEEE Trans. Pattern Anal. Mach. Intell..

[35] Cao P., Liu X., Yang J., Zhao D., Huang M., Zhang J., Zaiane O. (2017). Nonlinearity-aware based dimensionality reduction and over-sampling for AD/MCI classification from MRI measures. Comput. Biol. Med..

